# Towards a rational design of faecal transplant analogues

**DOI:** 10.1038/s41598-019-42043-x

**Published:** 2019-04-03

**Authors:** Olaf F. A. Larsen, Anton H. J. Koning, Peter J. van der Spek, Eric Claassen

**Affiliations:** 1 Vrije Universiteit Amsterdam, Athena Institute, De Boelelaan 1085, 1081 HV Amsterdam, The Netherlands; 2 Erasmus Medical Centre Rotterdam, Department of Pathology, Clinical Bioinformatics Unit, Dr. Molewaterplein 40, 3015 CD Rotterdam, The Netherlands

## Abstract

Faecal transplants (microbiota transfer) have shown to be promising therapies having a wide range of therapeutic applications. However, current safety considerations hamper further valorisation. As such, well designed faecal transplant analogues provide an interesting alternative to minimize possible safety aspects. However, to date little knowledge on how to rationally design such analogues exists. Here, we show by applying first order basic graph theory that such analogues dedicated to restoring a specific physiological functionality (a microbial guild) should consist of 5–6 species to maximize stability, efficiency, and minimize safety issues and production costs.

## Introduction

The human microbiota has triggered tremendous interest as its composition is linked to health and disease^1^. Consequently, a human individual must nowadays be considered as an ecosystem comprising not only the human cells but also the various microbiotas linked to virtually all body sites ranging from the skin, genitals, to the gastro-intestinal tract^1^. The gastrointestinal microbiota has specifically drawn attention, as a large collection of research articles indicates a role of the gut microbiota to a variety of illnesses such as obesity, diabetes, autism and Alzheimer’s disease^2^. This role can be narrowed down to the (mal)functioning of dedicated physiological functionalities, reflecting small microbial ecosystems. An example of this is the production of short chain fatty acids in type 2 diabetes mellitus^3^. Although alterations in the gut microbiota and the onset of disease are often still associations, there is for many cases increasing evidence that a dysbiosis of the gut microbiota indeed is (one of the) initial causes that actually leads to disease^4^. Hence, modulation of the gut microbiota, for example by pre-and probiotics, opens a window of opportunity for both disease prevention and management^5^. Faecal transplant analogues provide as such a probiotic intervention *in extremo*, by administering donor faeces from a healthy subject to a diseased subject, thereby effectively “repoopulating” the entire gastrointestinal tract with a new microbiota^6^. Although spectacular results have been obtained for various conditions such as *Clostridium difficile* infections^7^, insulin insensitivity^8^, or even autism^9^, safety considerations remain and hamper the valorisation of these types of intervention^10^. Hence, it would be desirable to ultimately develop (personalized) faecal transplant analogues that provide the necessary microbial ecosystem tailored to rebalance a specific physiological functionality within the human body and thereby effectively clearing the associated disease state. In such a way, by providing a well-defined and minimal ecosystem, safety can ultimately be guaranteed. To understand and rationally design such transplant analogues (whether these consists of bacterial species or, as more recently suggested, bacteriophages^11^), one should have a thorough understanding of the microbial network and corresponding signalling pathways that provide the desired physiological effect. Therefore, as a first step, we recently modelled small microbial guilds using elementary graph theory^12^. Microbial guilds are small ecosystems tailored to a single functionality^13^, which are known to exist in the gut microbiota^14^. Our calculations indicated that the functional efficiency of microbial guilds gets higher by introducing more species. Furthermore, redundancy in functional efficiency takes place after the diversity of species is sufficiently high. These simulations were performed by making use of undirected graphs, meaning that the communication between the microbial entities was either existing (in two-ways) or absent. However, microbial interactions are known to be directed^15^. Therefore, this article presents simulations on directed microbial guilds. As such, the calculations provide the complete configurational landscape possible between the two extremes: only directed interactions and only undirected interactions. Also, the efficiency of usage of the building blocks for setting up all configurations to construct actual signalling pathways was calculated. By combining these results, a window of opportunity for the future development of faecal transplant analogues could be constructed. This window shows that future faecal transplant analogues tailored to restore a single specific functionality should consist of 5 to 6 microbial species.

## Methods

In a microbial guild, an initial species gets triggered by some external factor like a dietary component, antibiotics or stress. This trigger is then signalled to a subsequent species and ultimately transported to the “target” species that produces the associated physiological response. In our simulations we consider species number 1 as the starting species and species number *n* (e.g. species number 5 for a guild consisting of *n* = 5 species) as the target species.

**Figure 1:**
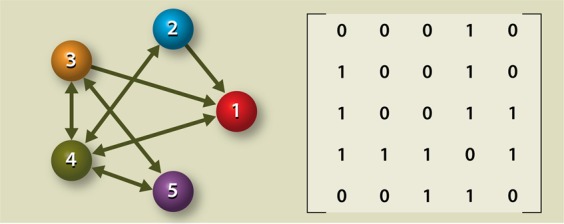
Graph theoretical representation of a hypothetical microbial guild consisting of 5 different species (represented by the nodes). The communication between the species is depicted by the arrows (edges). In this example, species 1 cannot exert a signal to species 2, but the other way around is possible. Communication between species 3 and 4 is possible in both directions. The associated adjacency matrix is also shown. This matrix shows all connections going from species *n* (the row numbers) to species *m* (the column numbers), by either displaying a “1” (directed connection from *n* to *m*) or “0” (no directed connection). In this example, species 1 would be the starting node receiving the initial trigger, whereas species 5 would be the target node producing the associated physiological response. One of the possible signalling pathways could be: node 1 → node 4 → node 5.

We modelled hypothetical microbial guilds using elementary network theory. As such, a microbial guild can be depicted by a graph, in which the nodes represent the microbial species and the arrows (edges) the directed communication channels between the microbial species (see Fig. 1).

All graphs we modelled were unweighted, meaning that all communication channels have the same signalling strength. Also, the graphs did not contain loops or multiple edges between two species. Only simple paths were calculated, hence every node can only be visited once maximally.

To get the total signalling landscape possible, we calculated for a hypothetical guild comprising of *n* species all possible configurations (adjacency matrices). For each adjacency matrix, the density was also calculated. The density *D* of an adjacency matrix (configuration) is defined as: $D=\frac{E}{m}$, with *E* being the number of edges present for the specific configuration investigated, and *m* the maximum number of edges possible for the number of nodes given.

For each adjacency matrix, all paths from species 1 to species *n* were calculated. This exercise was performed for hypothetical systems ranging from *n* = 2 to *n* = 5 species. For each set of nodes (ranging from *n* = 1 to *n* = 5), we constructed a heatmap depicting the number of paths from node 1 to *n* as a function of the density *D* of the adjacency matrix and the number of steps (edges travelled) needed to go from species 1 to species *n*.

## Results

**Figure 2:**
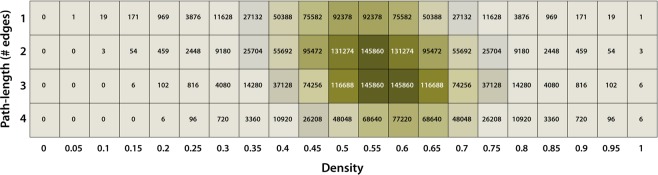
Heatmap depicting the number of paths for all configurations (directed adjacency matrices) possible for 5 species, as a function of the density and the number of steps needed to go from species 1 to species 5 (pathlength).

In Fig. 2, the heatmap for *n* = 5 is depicted as an example of all the heatmaps we calculated. For 5 nodes, the number of adjacency matrices already equals 1.048.576, and the total number of paths to go from species 1 to 5 equals 2.490.368.

**Figure 3:**
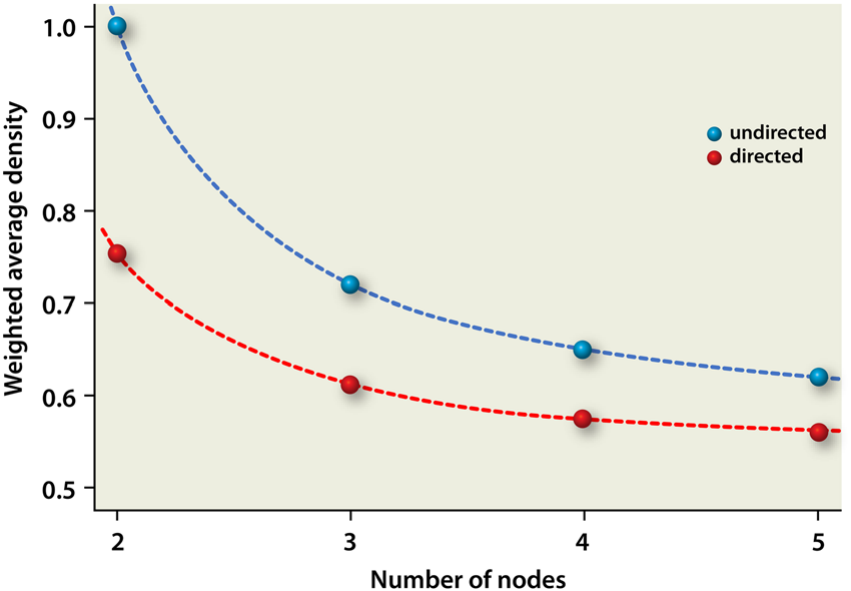
Weighted average density as a function of the number of nodes. Blue: undirected graphs. Red: directed graphs. The results for undirected interactions were already obtained earlier and are plotted here for completeness^12^.

Analogous to our previous calculations^12^, we plot the weighted average density as a function of the number of nodes. For comparison, we plot both the results earlier obtained for undirected graphs^12^ as well as the current results for directed graphs, see Fig. 3. As such, the configurational space between the two extremes (only directed and only undirected interactions) can be depicted.

**Figure 4:**
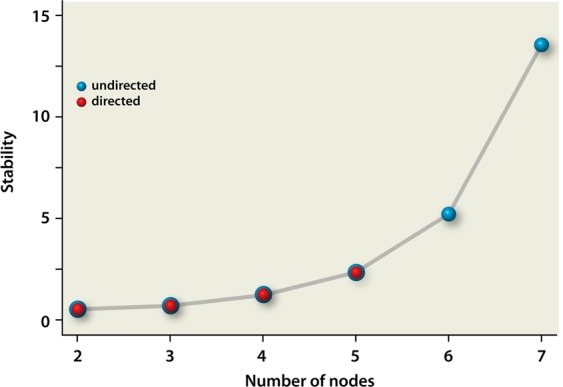
Stability as a function of nodes (species). The stability is defined as the total number of paths divided by the total number of adjacency matrices. The red dots represent the results for the directed configurations, that overlap with those for the undirected configurations (not shown for *n* = 1–5). For completeness, the undirected stability is also shown for 6 and 7 nodes (blue dots). The line is added as a guide to the eye. Please note that the results for the undirected graphs were already obtained earlier and are plotted here for completeness^12^.

Subsequently, we calculated the stability, represented by the ratio of the number of paths and adjacency matrices. The stability increases with the number of nodes for both directed and undirected networks. Interestingly, there is no difference between undirected and directed networks (see Fig. 4).

**Figure 5:**
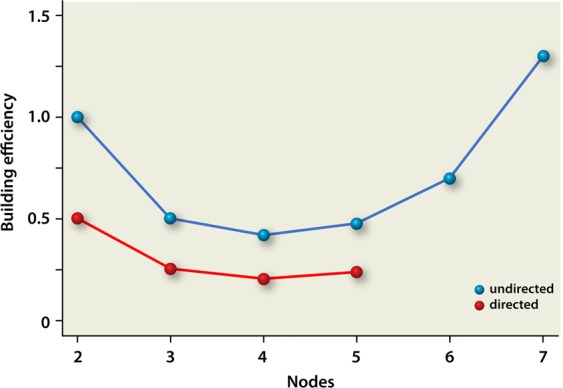
Building efficiency as a function of nodes. Note that the building efficiency is calculated up to 5 nodes for directed graphs due to computational constraints. The minimum at *n* = 4 and the upward trend after *n* = 4 like for the undirected configurations is, however, also still clearly visible for the directed configurations. Please note that the building efficiency for undirected interactions is calculated using results we obtained earlier^12^.

Finally, we calculated a metric we call the “building efficiency”. This efficiency was calculated as follows. We first summed the numbers of paths for all cells of a heatmap for a specific number of nodes (*n*). This results in all paths possible for going to node 1 to node *n* originating from all configurations possible (note that identical paths will be summed numerous times, we just calculate the total number of paths and not the number of unique paths). Subsequently, we calculated the total number of edges necessary for the construction of all configurations possible at that specific number of nodes *n* (note that this sum will also contain edges originating from configurations that do not result in a path from 1 to *n* at all). As an example, for *n* = 5, using directed edges, the total number of paths equals 2.490.368, and the total number of edges equals 10.485.760. Finally, we calculate the ratio of all these paths and edges (number of paths divided by number of edges), which equals for *n* = 5 (using directed edges) to 2.490.368/10.485.760 = 0.2375. In Fig. 5, we plotted this building efficiency as a function of nodes for both directed and undirected graphs.

## Discussion

As can be seen from Fig. 3, for both undirected and directed networks, the weighted average density decreases upon introducing more species (nodes). This implies that the maximum signal that can be obtained (highest number of paths from node 1 to node *n*) corresponds to configurations having lower density upon increasing the number of nodes. Hence, it is from a “design (evolutionary) point of view” advantageous to have more nodes (more diversity) because one can then construct on average networks that still produce the highest signal while requiring relatively less interactions for a node with other nodes. Our simulations show that, upon going from undirected to directed interactions, this trend is also present, surprisingly, at very low densities. Hence, directed interactions result in more efficient systems as compared to undirected interactions. We hypothesize that this result, improved efficiency, provides a mathematical rationale why microbial interactions in small ecosystems are found to be directed in nature.

A second feature that can be obtained from our simulations and has been discussed earlier^12^, is that the weighted average density levels off at relatively higher numbers of nodes. This feature has been explained in terms of redundancy: when surpassing a minimum number of nodes, the total efficiency will not improve anymore (or just slightly). This asymptotic trend reflects robustness of the system: species can be taken out of the system without significantly reducing its efficiency. As can be seen from our calculations, it seems that such redundancy takes place earlier in ecosystems consisting of (only) directed interactions, starting already at ~5 nodes, as compared to ecosystems consisting of (only) undirected interactions (starting at ~6 nodes^12^). Hence, directed configurations are more efficient than undirected configurations.

The fact that the stability as a function of nodes does not change upon switching from undirected to directed interactions, implies that one can obtain a higher efficiency (weighted average density) at a specific number of nodes when using directed instead of undirected interactions, while still having the same stability. Hence, also this result advocates the (evolutionary) advantage of directed microbial interactions as compared to undirected interactions.

To further investigate plausible evolutionary drivers for the construction of small ecological systems like microbial guilds, we calculated a ratio called the building efficiency (see results section, Fig. 5). As such, this ratio provides us with information on the efficiency of usage of the building blocks provided in an evolutionary process. In other words: for a fixed number of nodes, when one can utilize all evolutionary building blocks (the edges) needed to construct all possible configurations for the number of nodes provided, this number provides the “building efficiency” expressed as the number of actual paths for node 1 to node *n* per edge.

Strikingly, the building efficiency goes down when starting from two nodes up to a minimum at 4 nodes, and then goes up again. This building efficiency is overall lower for directed interactions with respect to undirected interactions but displays the same trend. The shape of these curves strongly resembles a so called “smiling curve” which has been described earlier for manufacturing processes^16^. The smiling curve for manufacturing processes shows how added value varies within different stages of a production process. In the early stages of the production process (R&D phases), added value is going down, whereas added value is going up again at later stages (e.g. the phase when the product is launched, and marketing is key). Our results can be explained in a similar fashion. Construction of microbial guilds in “early stages” (not enough nodes yet), results in poor added value (and an initial decline of value when more nodes are added). However, when the number of nodes reaches a critical point, the microbial guilds constructed are getting mature and will gain in added value (in our simulations: building efficiency). This explanation is supported by the fact that stability only starts to significantly rise after 4 nodes, and that redundancy also comes into play after 5 nodes. Consequently, being two sides of the same coin, loss of diversity gives problems.

**Figure 6:**
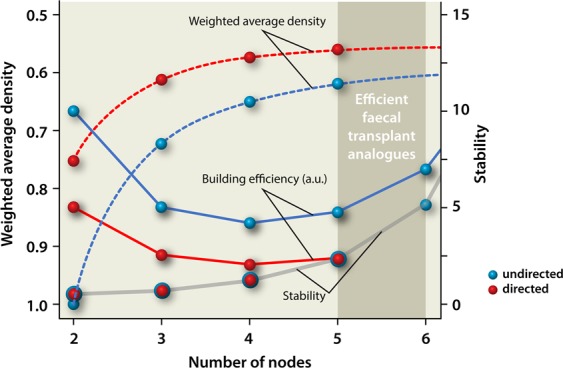
Graphical summary of our findings, providing the window of opportunity for the construction of faecal transplant analogues. The building efficiency is given in arbitrary units (a.u). See text for details. The results for undirected interactions with respect to weighted average density and stability were already obtained earlier^12^ and are duplicated here for completeness.

From these results, we have constructed a panel that summarizes our results and provides us with the window of opportunity for the construction of faecal transplant analogues, see Fig. 6.

As can be seen from Fig. 6, the window of opportunity for the construction of faecal transplants lies within the domain between 5 and 6 nodes. After 6 nodes, one will have still more stability, but redundancy has set in already at 6 nodes. Hence, from a cost and safety aspect, one would like to strive for a minimum number of microbial species. Hence, as such 6 different species provide the upper limit. From a building efficiency point of view, 4 nodes provide the lower limit, because from that number of nodes the building efficiency (added value) is starting to rise. However, redundancy has not set in yet at 4 nodes, and at 4 nodes the stability is also still very limited. Redundancy does set in at 5 nodes for directed interactions, which is the type of interactions utilized in nature (contrary to undirected interactions, where redundancy sets in at 6 nodes^12^). Hence, by combining all aforementioned considerations, 5–6 nodes seem to be optimal for the construction of faecal transplant analogues.

Interestingly, the building efficiency is structurally lower for directed interactions as compared to undirected interactions. At a first glance, this result would plead for usage of undirected interactions when constructing faecal transplant analogues. However, one should realize that the overall building efficiency as depicted here is built up from all possible configurations and should be envisioned in an evolutionary perspective. From a rational design point of view, however, only one configuration will eventually be utilized as the faecal transplant analogue. As such, the smiling curves only provide us with the minimum number of nodes needed to have an “evolutionary process” in the laboratory when seeking for a feasible faecal transplant analogue to be efficient.

It is interesting to note that this perspective to envision physiological functionalities within the gut microbiota provides us with arguments pleading for the usage of both single-strain as well as multi-strain probiotic preparations. When a microbial guild is misfunctioning (dysbiosis) due to the absence of only one species within the guild, application of the proper single strain probiotic could be sufficient to restore its functionality. When more species are absent or when the whole guild is absent, application of the proper multi-strain preparations (up to a faecal transplant analogue in case all species are absent) could restore the functionality of the guild. As an example, one could envision repairing a microbial guild responsible for maintaining the integrity of the epithelial cell layer of the gut. Loss of this integrity would result in a “leaky gut”, resulting in penetration of lumen contents into the body that could cause inflammation.

It is important to mention that all the simulation results presented here are generic, although they are here being discussed within the framework of microbial guilds residing in the human gastrointestinal tract. Being generic results, they can also be used for microbial communities in, for example, soil, water, or fermented foods. Bioremediation could be a possible application area as well, provided that the removal of the toxic compounds can indeed be achieved by a relatively small ensemble (guild) of collaborating microorganisms.

In short, we have shown by first principle graph theory that the window of opportunity for the construction of faecal transplant analogues lies optimally between 5–6 microbial species. These results should be the starting point for the rational design of probiotics, ranging from single strain up to complete faecal transplant analogues.

## Data Availability

The datasets generated during and/or analysed during the current study are available from the corresponding author on reasonable request.
